# Development of gut microbiota and bifidobacterial communities of neonates in the first 6 weeks and their inheritance from mother

**DOI:** 10.1080/19490976.2021.1908100

**Published:** 2021-04-13

**Authors:** Bo Yang, Mengfan Ding, Yingqi Chen, Fengzhen Han, Chunyan Yang, Jianxin Zhao, Patrice Malard, Catherine Stanton, R. Paul Ross, Hao Zhang, Wei Chen

**Affiliations:** a State Key Laboratory of Food Science and Technology, Jiangnan University, Wuxi, China; b School of Food Science and Technology, Jiangnan University, Wuxi, China; c International Joint Research Laboratory for Pharmabiotics & Antibiotic Resistance, Jiangnan University, Wuxi, China; d Department of Gynaecology and Obsterics, Guangdong Province People’s Hospital, Guangdong Academy of Medical Science, Guangzhou, China; e National Engineering Research Center for Functional Food, Jiangnan University, Wuxi, China; f Biostime (Guangzhou) Health Products Ltd., Guangzhou, China; g Food Bioscience, Teagasc Food Research Centre, Fermoy, Ireland; h APC Microbiome Ireland, University College Cork, Cork, Ireland; i Wuxi Translational Medicine Research Center and Jiangsu Translational Medicine Research Institute Wuxi Branch, Wuxi, China

**Keywords:** Gut microbiota, bifidobacterial communities, diversity, transmission, infants

## Abstract

Microbiota especially *Bifidobacterium* play an important role in adjusting and maintaining homeostatic balance within the infant intestine. The aim of this study was to elucidate the relationship between maternal and infant gut microbiota and identify the *Bifidobacterium* species that may transfer from mother to infant over the first 42 days of the infant’s life. Nineteen mother-infant-pair fecal samples were collected and the diversity and composition of the total bacterial and *Bifidobacterium* communities were analyzed via 16S rDNA and bifidobacterial *groEL* gene high throughput sequencing. The results revealed that the relative abundance of *Bifidobacterium* was significantly higher in the infant gut while *Parabacteroides, Blautia, Coprococcus, Lachnospira* and *Faecalibacterium* were at lower relative abundance in 7-day and 42-day infant fecal samples compared to the maternal samples. The maternal gut has more *B. pseudocatenulatum*. In the infant group, *B. breve* and *B. dentium* relative abundance increased while *B. animalis* subsp. *lactis* decreased from days 7 to 42. Additionally, *B. longum* subsp. *longum* isolated from FGZ16 and FGZ35 may have transferred from mother to infant and colonized the infant gut. The results of the current study provide insight toward the infant gut microbiota composition and structure during the first 42 days and may help guide *Bifidobacterium* supplementation strategies in mothers and infants.

## 1. Introduction

The infant gut microbiota plays a key role in immunity development of its host with both immediate and long-term health effects.^1^ The mother’s microbiota (from intestine during vaginal delivery and breast milk during feeding) seed the infant intestine first, thus influencing and selecting for the microbiota that follow, leaving a footprint that can be detected and even continued into adulthood.^2,3^ Studies have already highlighted the critical role of mother-derived infant gut microbiota.^4^ Indeed, by analyzing the microbiota profile in human breast milk^5^ and infant feces^6^ scientists found that microbes transfer from the maternal gut to the mammary glands through an entero-mammary pathway and subsequently colonize the infant intestine.^7^ Despite this, only a limited number of bacterial strains have been proven to be shared between mother-infant pairs.^8,9^

Bacterial diversity in the infant gut increases with age in the first 12 months and the complex ecological network that forms during the first 6 months of life is built on complex microbiota ecosystem.^6^ Actinobacteria, specifically *Bifidobacteriaceae*, increase as the infant grows and thus members of *Bifidobacteriaceae* are dominant in the infant gut.^6^ *Bifidobacterium*, one of the first colonizers, is the dominant bacterium in breastfed infants and has important effects on the development of the gut microbiota and subsequent physiological state and infant health.^10,11^ A number of studies have focused on the development of bifidobacteria in premature infants,^12^ those born by Cesarean section,^13^ formula fed and from different geographical locations.^11^ Fewer studies have paid attention to the specific relationship between the maternal and infant gut microbiota, especially *Bifidobacterium*, and post-birth development until one-month of age. Additionally, quantifying the extent to which *Bifidobacterium* from mother shapes and colonizes the infant gut in early childhood has remained a difficult challenge in the field of microbial research.^14^ Therefore, the current study aimed to characterize the early development of the gut microbiota and *Bifidobacterium* communities in the infant gut by 16S rRNA and bifidobacterial GroEL, respectively, based on high-throughput sequencing approaches, and to identify the potential transmission of *Bifidobacterium* at strain level from mothers to their corresponding infants via isolation and comparative genomic analysis.

## 2. Methods

### 2.1 Subject recruitment and sample collection

This study was approved by the Ethical Committee of Guangdong People’s Hospital, Guangdong Academy of Medical Sciences (No. GDREC2017183H (R1)), Guangzhou, China. Informed written consent was obtained from all the participants or their parents. Nineteen mother-infant pairs were recruited at Guangdong People’s Hospital during June 2017 to September 2018. All the recruits were considered to be healthy based on self-reporting. All the infants were full-term, vaginally delivered and breastmilk fed. Maternal stool samples were collected during the last week of pregnancy, and infant stool samples were collected at two different time points, 7-days and 42-days after birth. Samples were immediately frozen at −20°C and delivered with dry-ice to the laboratory within 2 hours.

### 2.2 Fecal genomic DNA extraction and Illumina sequencing

Genomic DNA in stool samples was extracted using FastDNA® Spin Kit (MP Biomedicals, Santa Ana, CA) according to the manufacturers’ instructions. The microbiota composition of the samples were established by amplicon sequencing of a ~ 500bp fragment of 16S rRNA V3-V4 region through Illumina MiSeq sequencing. PCR was performed using primers 341 F (5ʹ-CCTAYGGGRBGCASCAG-3ʹ) and 806 R (5ʹ-GGACTACNNGGGTATCTAAT-3ʹ) as previously described.^15^ Each PCR reaction (50 μL) contained 25 μL Taq Master Mix (2×), 1 μL genomic DNA, 1 μL 341 F (20 μM), 1 μL 806 R (20 μM) and 22 μL ddH_2_O. The PCR conditions were as follows: initial denaturation at 95°C for 5 min, followed by 30 cycles of 95°C for 30 s, 52°C for 30 s and 72°C for 30 s, and a final extension of 72°C for 7 min. Assessment of *Bifidobacterium* species was carried out as described previously.^16^ Partial bifidobacterial *GroEL* gene (Bif-GroEL) fragment (~500 bp) was amplified using primers Bif-*groEL*-F (5ʹ-TCCGATTACGAYCGYGAGAAGCT-3ʹ) and Bif-*groEL*-R (5ʹ-CSGCYTCGGTSGTCAGGAAC-AG-3ʹ). The PCR reaction (50 μL) contained 25 μL Premix Taq Master Mix (2×), 2 μL genomic DNA, 1 μL Bif-*groEL*-F (20 μM), 1 μL Bif-*groEL*-R (20 μM) and ddH_2_O 21 μL. The PCR procedures were as follows: pre-denaturation at 95°C for 5 min, followed by 30 cycles consisting of denaturation at 95°C for 30 s, annealing at 60°C for 30 s and extension at 72°C for 1 min, and a final extension of 72°C for 10 min. All the PCR products were purified by QIAquick Gel Extraction Kit (QIAGEN, Hilden, Germany) and quantified using the Qubit^TM^ dsDNA BR Assay Kit (Life Technologies, Carlsbad, CA) according to the instructions. DNA amplicon libraries were prepared with TruSeq Nano DNA LT Kit (Illumina, San Diego, CA) and sequenced with the MiSeq Reagent Kit v3 (600 cycles-PE, Illumina, San Diego, CA) on the Illumina MiSeq platform following the instructions.

### 2.3 Sequence data processing and statistical analysis

The sequences reads were processed with QIIME 2 package (Quantitative Insights into Microbial Ecology, Flagstaff, AZ).^17^ The raw reads were screened as previously described.^18^ Only pair-end reads overlapping >10 bp and without any mismatch were assembled. Barcode and sequencing primers from the above assembled sequences were trimmed. The operational taxonomic units (OTU) were established *de novo* using UCLUST with 97% sequence identity cut off. The OTUs of the V3-V4 region were assigned by the Ribosomal Database Project (RDP) Naive Bayes classifier. The OTUs of Bif-GroEL sequences were assigned by *Bifidobacterium* GroEL database.

Py NAST aligner was applied to compare the sequences with SiLVA core set.^19^ The dilution curve, alpha diversity and beta diversity of the sample were performed by QIIME 2.^17^

### 2.4 Bifidobacterium isolation and identification

All the stool samples were assessed for the presence of *Bifidobacterium*. One gram of each stool sample was blended with 9 mL sterile physiological saline. Serial dilution and plating were executed in an anaerobic workstation (AW400TG, Electrotek Scientific Ltd., West Yorkshire, UK). 100 μl of diluent was continuously plated on de Man-Rogosa-Sharpe (MRS) agar plus 0.05% (w/v) L-cysteine hydrochloride (mMRS), 100 mg/L mupirocin (Sangon Biotech Co., Ltd., Shanghai, China) and 50 U/mL nystatin (Sangon Biotech Co., Ltd., Shanghai, China). Agar plates were cultured in the anaerobic workstation flushed with 80% N_2_, 10% CO_2_, and 10% H_2_ at 37°C for 72 h. For each sample, colonies on mMRS plates were counted. Colonies were selected at random and re-streaked onto mMRS agar for purity. The final pure culture was cultured in mMRS broth and preserved in 30% glycerol at −80°C. DNA was extracted from each strain using the Rapid Bacterial Genomic DNA Isolation Kit (Sangon Biotech Co., Ltd., Shanghai, China) and stored at −20°C. Each of the putative *Bifidobacterium* isolates was identified by a 16S rRNA sequence using the bacterial universal primers (27 F: 5ʹ-AGAGTTTGATCCTGGCTCAG-3ʹ and 1492 R: 5ʹ-ACGGCTACCTTGTTACGACTT-3ʹ) by BGI (Shenzhen, China). All the strains were compared with the NCBI BLAST database (http://www.ncbi.nlm.nih.gov/BLAST/) to assign a particular species.

### 2.5 Bifidobacterial genome sequencing and bioinformatics analysis

All the bifidobacterial isolates from mother-infant pairs were draft-genome sequenced using an Illumina Hiseq×10 platform (Majorbio BioTech Co., Ltd, Shanghai, China), with the use of 2 × 150 bp paired-end libraries (average read length of about 400 bp). The assembly was performed using SOAPdenovo v2.04 software,^20^ and the partial gap was filled by the software GapCloser.^20^ Bacterial gene prediction was performed using Glimmer 3.02 software and GeneMarkS v4.30 software.^21^ Open Reading Frames (ORF) were predicted via a combined method of the predictor Prodigal v2.0 (http://prodigal.ornl.gov).^21^ BLASTX v2.2.26 alignment was carried out for all the genomes assayed^22^

All-versus-all BLASTP alignment (50% identity; e-value 1e-4 cutoff) was executed for protein sequences extracted from each strain.^23^ BLASTP outputs were used as inputs to cluster proteins into families sharing the same function using the Markov Cluster Algorithm (MCL) with an inflation index of 2.5.^24^ The gene families obtained were classified to the core genome or the dispensable genome, based on their existence in all or in a subset of the strains investigated. To calculate ANI values for each pair of genomes, an ANI Perl script was implemented (https://github.com/chjp/ANI/blob/master/ANI.pl).^25^

All the genomes were annotated using the HMMSCAN software^26^ The carbohydrate active enzyme gene annotation profiles were obtained and compared using the carbohydrate-active enzymes (CAZy) database (http://www.cazy.org/).^27^ All the genomes were annotated with the comprehensive antibiotic research database (CARD) (https://card.mcmaster.ca/home) to obtain information of predicted antibiotic resistance genes encoded by each genome.^28^ Cluster analysis was performed using HemI software.^29^

### 2.6 Statistical analysis

Significant differences among different groups were analyzed by paired sample mean test and calculated by SPSS 25.0.

## 3 Results

### 3.1 Gut microbiota development of infants in the first 6 weeks

To characterize the gut microbiota composition in the infant gut, fecal samples from 19 infants were collected at days 7 and 42 after birth. The maternal fecal samples were also collected during the last week of pregnancy. The microbiota composition of all fecal samples was analyzed by 16S rRNA V3-V4 region high-throughput sequencing. In total, those 57 samples yielded 2,040,477 reads, ranging from 8,619 to 133,129 reads. Reads were classified into OTUs at a 3% similarity cutoff, and generated 548 ~ 7,271 OTU per sample.

**Figure 1.**
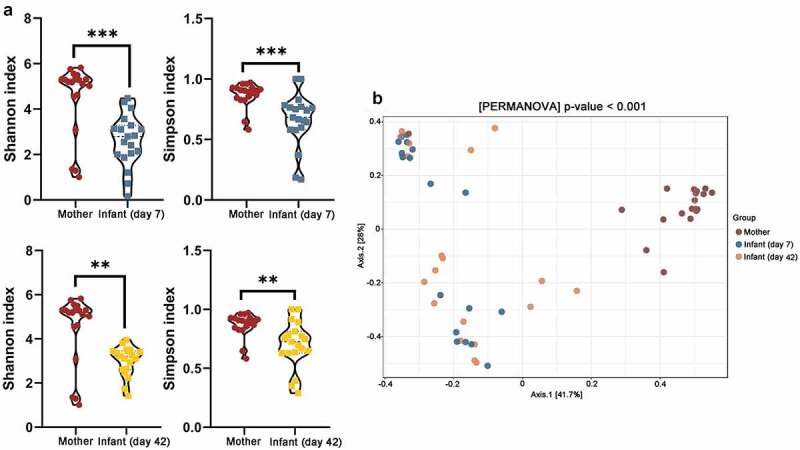
Microbiota diversity and composition in maternal and infant gut microbiota. (a) Alpha diversity and (b) Beta diversity of mothers and infants gut microbiota; **, *p* < .01, ***, *p* < .001. Significant difference of Beta diversity was calculated by PERMANOVA using Bray-Curtis Index of three groups

Alpha diversity indices were used to estimate the microbial richness. Maternal feces showed significant higher diversity compared to that in 7-day-infant gut (*p* < .001) and 42-day-infant gut (*p* < .01, Figure 1(a), Shannon index, group Mother: 4.50 ± 0.86, group Infant (day 7): 2.62 ± 1.15, group infant (day 42): 3.00 ± 0.67; Simpson index, group Mother: 0.86 ± 0.09, group Infant (day 7): 0.65 ± 0.22, group infant (day 42): 0.70 ± 0.19). The similarities among the microbial communities were estimated using the principal coordinate analysis (PCoA).^30^ Bray-Curtis Index was used to compared distance among groups and PERMANOVA was used to calculated difference. The samples were clustered to observe the structural similarity of the species. PCoA indicated that an obvious separation of microbiota composition existed between maternal and infant samples but there was no discrimination between infant samples at different time points (Figure 1(b)).

**Figure 2.**
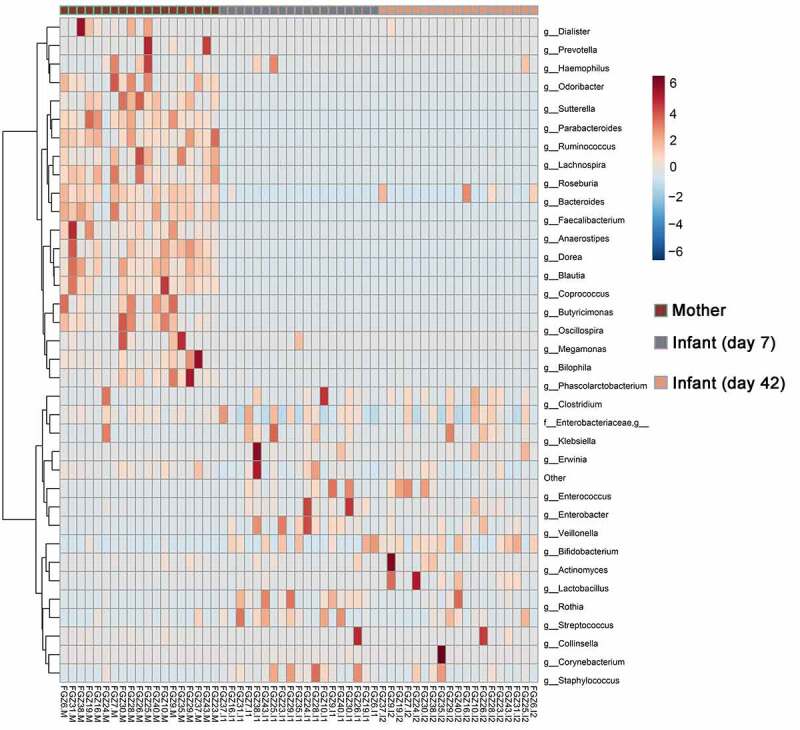
Composition of infant and maternal gut microbiota at genus level. The corresponding value of color is the value of the relative abundance of each genus after row normalization. Red means higher relative abundance and blue means low relative abundance

At genus level, *Parabacteroides, Ruminococcus, Lachnospira, Roseburia, Bacteroides* and *Faecalibacterium* were the dominant genera in the maternal gut. Unclassified *Enterobacteriaceae, Bifidobacterium, Streptococcus, Enterococcus, Staphylococcus, Rothia* and *Veillonella* appeared to be predominant in the 7-day-infant gut, while Unclassified Enterobacteriaceae, *Bifidobacterium, Streptococcus, Bacteroides, Clostridium, Enterococcus, Staphylococcus* and *Lactobacillus* were the predominant genera in the 42-day-infant gut (Figure 2). Additionally, some genera were present in greater relative abundance in infant samples compared with maternal samples including *Enterobacter, Bacillales*, Unclassified Planococcaceae, *Actinetobacter, Corynebacterium, Rothia, Pseudomonas, Propionibacterium, Allobaculum, Enterococcus, Bifidobacterium, Lactobacillus, Klebsiella, Streptococcus, Bacteroides* and *Staphylococcus*.

### 3.2 Differences between mother gut and infant gut

**Figure 3.**
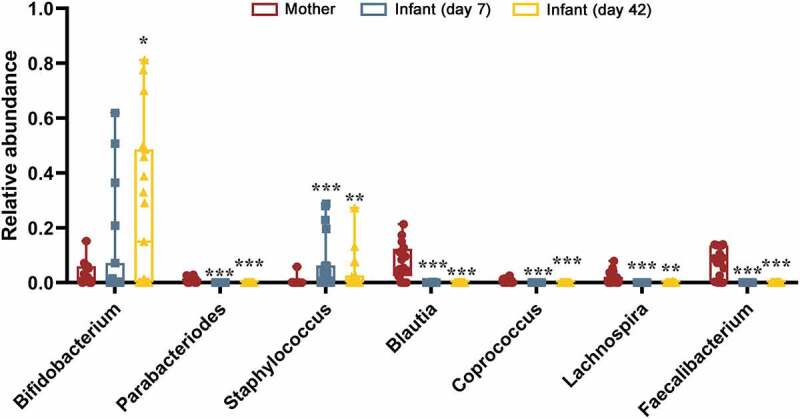
Discriminative genera among the three groups. All results were compared to maternal gut group; *, *p* < .05; **, *p* < .01; ***, *p* < .001

To investigate the typical communities or species in sample partitioning, LDA EffectSize (LEfSe analysis) was conducted with linear judgment (LDA) to estimate the typical bacteria in each group (Figure S1). Three genera were discriminated in infants at 7-days after birth, with *Staphylococcus* having the greatest discriminatory power, while infants at 42-days were discriminated by higher *Achromoacter* (Alpha value = 0.05, LDA score = 2.0). When comparing the infant samples at 7-day and 42-day with mother samples, respectively, 7-day infant was discriminated by higher Unclassified *Enterobacteriaceae* and lower *Bacteroides* (Alpha value = 0.01, LDA score = 5.0); while 42-day infant was discriminated by higher *Bifidobacterium* and lower Unclassified *Lachnospiraceae* (Alpha value = 0.01, LDA score = 5.0, Figure S1). Furthermore, maternal samples appeared to have greater relative abundance of *Parabacteroides, Blautia, Coprococcus, Lachnospira* and *Faecalibacterium* compared to infant samples (*p* < .001) but less *Bifidobacterium* compared to 42-day infants (*p* < .05) and less *Staphylococcus* compared to 7-day (*p* < .001) and 42-day infants (*p* < .01) (Figure 3).

### 3.3 Diversity of the bifidobacterial community at species level in infants in the first 6 weeks

To characterize the *Bifidobacterium* community at species level in the infant gut, a recently developed strategy based on high-throughput sequencing of a hypervariable *GroEL* region was employed. GroEL profiling of all the infant samples generated a total of 635,030 reads, ranging from 5,356 to 27,785 reads per sample, which were grouped into clusters of identical sequences and then taxonomically classified. Eleven samples (FGZ24I1, FGZ16I1, FGZ25I, FGZ10I1, FGZ26I1, FGZ28I1, FGZ19M, FGZ26I2, FGZ29I2, FGZ28I2) did not show the composition of *Bifidobacterium* at species level due to low abundance of *Bifidobacterium* at genus level. Among these samples, FGZ19M was maternal fecal sample; FGZ24I1, FGZ16I1, FGZ25I and FGZ10I1 were 7-day-infant fecal samples; and FGZ29I2 was a 42-day-infant fecal sample. In addition, FGZ26I1 and FGZ26I2, FGZ28I1 and FGZ28I2 were from the same corresponding infant but at two time points, respectively.

**Figure 4.**
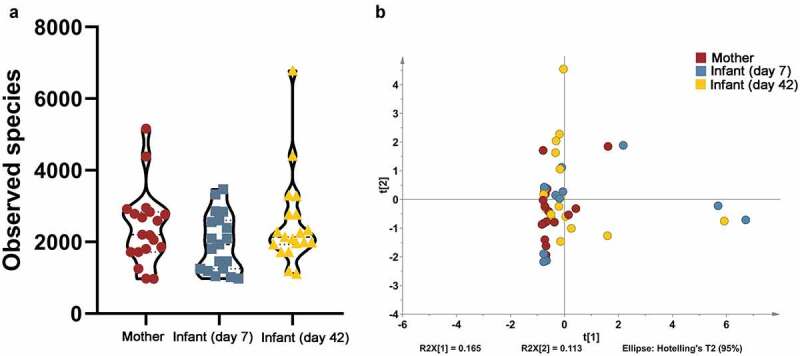
Diversity of *Bifidobacterium* in mother-infant pairs. Observed species of *Bifidobacterium* (a) and Beta diversity of *Bifidobacterium* (b)

**Figure 5.**
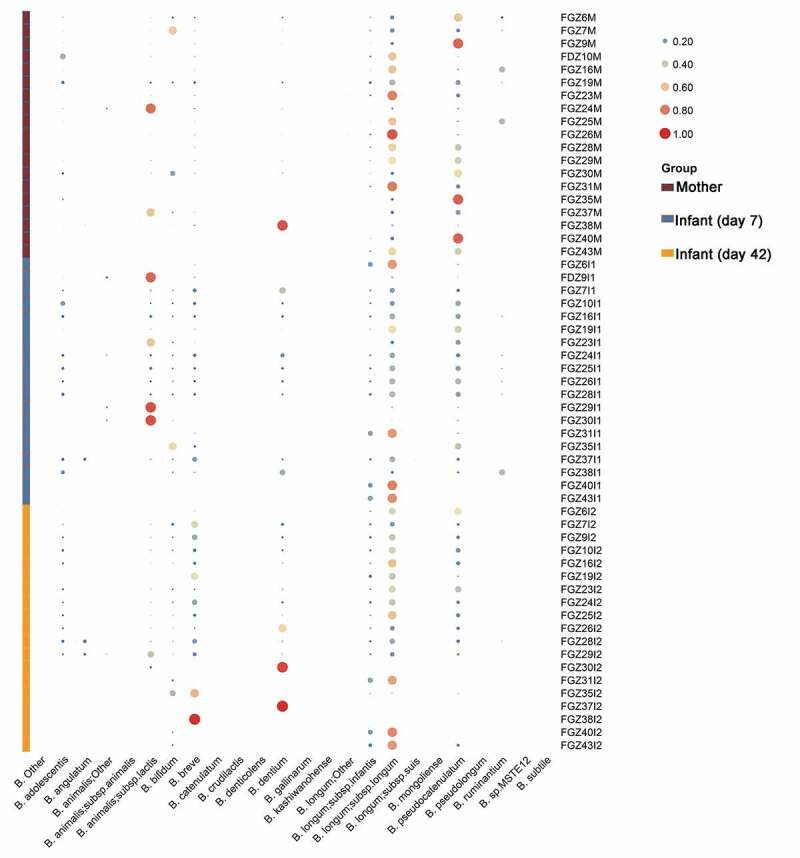
*Bifidobacterium* profiles in mother-infant pairs. The size of bubble represents the relative abundance of *Bifidobacterium* in each sample

The alpha diversity of samples was calculated based on the GroEL-based OTU results. No significant differences were found in observed species among 7-day infant, 42-day infant and maternal samples (Figure 4(a)). Additionally, the PCA results revealed that samples did not cluster based on the individual groups (Figure 4(b)). OTU analysis revealed that the dominant *Bifidobacterium* in maternal and infant samples were *B. longum* subsp. *longum* (34.6%±25.8%), *B. pseudocatenulatum* (19%±21.7%), *B. animalis* subsp. *lactis* (10.2%±23.9%), *B. breve* (9.2%±17.2), *B. dentium* (9.0%±22%), *B. longum* subsp. *infantis* (5.2%±6.7%), *B. bifidum* (4.5%±11.0%) and *B. adolescentis* (3.7%±5.5%) (n = 57, Figure 5). The relative abundance of *B. pseudocatenulatum* in the infant gut decreased compared to maternal samples while the other species increased from 31.3% to 13.5%. Additionally, *B. breve* and *B. dentium* in the infant gut increased from days 7 to 42 (5.6% vs 19%, 6.5% vs 14.4%, respectively) while *B. animalis* subsp. *lactis* decreased (19.3% vs 3.8%, Figure 5).

### 3.4 Bifidobacterial strains shared between mother and infants

As no significant differences were observed at species level for the bifidobacterial communities, bifidobacteria from infant and maternal fecal samples were isolated and identified by 16S rRNA gene sequencing to further investigate the bifidobacterial strains shared between mother-infant pairs. We isolated Bifidobacterium in 19 mother infant pairs of our studies but only 38 isolates were confirmed belong to 5 mother infant pairs. These Bifidobacterium including *B. longum* subsp. *infantis, B. longum* subsp. *longum, B. breve, B. pseudocatenulatum, B. animalis* subsp. *lactis, B. dentium, B. adolescentis* and *B. bifidum* (Table 1). To identify potential transmission of *Bifidobacterium* strains from mother and subsequent colonization in the infant gut, ANI analyses were performed on infant-mother corresponding fecal samples, with a value of 99% indicating the same source. Among the sixteen paired-isolates, *B. longum* subsp. *longum* isolated from FGZ16 and FGZ35 mother-infant pairs were originally from the same source, and similar results were found for *B. pseudocatenulatum* isolated from FGZ16 and FGZ35 mother-infant-pair samples (Table 1).

**Table 1.** Genome features of the isolates

Isolates	Pair ID	Origin	Contigs	Size(Mb)	GC%	ORF	tRNA	ANI (%)	Result
*B. longum* subsp. *longum* FGZ6-M-M10	FGZ6	Mother	50	2.14	60.0	1890	54	98.66	Not same source
*B. longum* subsp. *longum* FGZ6-I-d7-M6	FGZ6	7-day infant	71	2.38	59.9	2189	77		
*B longum* subsp. *longum* FGZ16-M-M1	FGZ16	Mother	75	2.29	60.1	2113	55	99.98	Same source
*B longum* subsp. *longum* FGZ16-I-d7-M5	FGZ16	7-day infant	82	2.27	60.1	2103	57		
*B longum* subsp. *longum* FGZ16-I-d42-M1	FGZ16	42-day infant	69	2.28	60.1	2107	58		
*B. pseudocatenulatum* FGZ16-M-M5	FGZ16	Mother	31	2.16	56.5	1933	53	99.98	Same source
*B. pseudocatenulatum* FGZ16-I-d7-M3	FGZ16	7-day infant	33	2.16	56.5	1916	56		
*B. pseudocatenulatum* FGZ16-I-d42-M6	FGZ16	42-day infant	34	2.16	56.5	1912	54		
*B longum* subsp. *longum* FGZ19-M-M7	FGZ19	Mother	42	2.31	59.8	1074	75	94.51	Not same source
*B longum* subsp. *longum* FGZ19-I-d7-M3	FGZ19	7-day infant	81	2.53	59.7	2506	57		
*B longum* subsp. *longum* FGZ35-M-M1	FGZ35	Mother	37	2.14	60.1	1913	62	99.97	Same source
*B longum* subsp. *longum* FGZ35-I-d7-M4	FGZ35	7-day infant	39	2.14	60.1	1915	58		
*B. pseudocatenulatum* FGZ35-M-M8	FGZ35	Mother	131	2.21	56.2	1994	56	99.89	Same source
*B. pseudocatenulatum* FGZ35-I-d7-M2	FGZ35	7-day infant	366	2.24	56.5	1949	64		

To further investigate if the bifidobacteria isolated from mother and infant pairs were shared, the genes involved in carbohydrate utilization were predicted by HMMER-3.1 and identified through the CAZY database along with antibiotic resistant genes by the CARD database. In total, 63 glycosyl hydrolase (GHs) families, ten glycosyl transferase (GTs) families, seven carbohydrate esterase (CEs) families, nine carbohydrate binding module (CBMs) families and one polysaccharide lyase (PLs) family were found among those strains. The heat-map of predicted carbohydrate utilization genes (Figure S2) shows that *B. longum* subsp. *longum* isolated from FGZ16 and FGZ35 possessed highly identical carbohydrate utilization genes. Additionally, 57 antibiotic resistance genes related to mupirocin, fosfomycin, kirromycin, fluoroquinolones, para-aminosalicylic acid, beta-lactam, fusidic acid, sulfonamides and aminocoumarin were found (Figure S3). The heat-map of predicted antibiotic resistance genes was constructed and indicated that *B. longum* subsp. *longum* isolated from FGZ6, FGZ16 and FGZ35, respectively, showed high similarity in their antibiotic resistance genes.

## 4. Discussion

To analyze early gut microbiota development and potential microbiota correlations between infants and their mothers, the diversity and composition of both gut microbiota and bifidobaterial communities of 19 mother-infant pairs were compared. At genus level, Unclassified *Enterobacteriaceae, Bifidobacterium Streptococcus Parabacteroides, Blautia, Coprococcus, Lachnospira* and *Faecalibacterium* showed significant different relative abundance in maternal faces, infant (day 7) faces, and infant (day 42) faces. At Bifidobacterium species level, *B. pseudocatenulatum was higher in maternal faces*. The relative abundance of *B. breve, B. dentium*, and *B. animalis* subsp. *lactis were significant different between* day 7 and day 42. Additionally, *B. longum* subsp. *longum* isolated from FGZ16 and FGZ35 respectively may transfer from mother to infant and colonized the infant gut.

Alpha diversity in the study illustrated that microbial richness in the maternal intestine was significantly higher than that in infants. The NIH research into the gut microbiota of healthy children and adults has shown that bacterial diversity increases with age in different populations.^31^ The results of PCoA indicated that the distance among infant samples was greater than that of maternal samples, showing a greater variance of gut microbiota among infants. So even though the gut microbiota of adults is more complex, the composition is relatively stable.^32^

The abundance of an unclassified *Enterobacteriaceae* and *Streptococcus* were higher in 7-day infants compared with 42-day infants. During the early establishment of the gut microbiota, the first microbial actors that render the gastro-intestinal environment fully anaerobic are facultative anaerobes, which include several members of the *Enterobacteriaceae* family.^33,34^ After the removal of oxygen, the infant intestine undergoes extensive colonization driven by strictly anaerobic bacterial taxa, such as those belonging to the genera *Bifidobacterium, Clostridium, Bacteroides* and *Ruminococcus*.^34,35^ Therefore, as intestinal oxygen decreases during the initial weeks after birth, the abundance of *Bifidobacterium* increases, which is reflected in the results of this study. Interestingly, the current results show that the relative abundance of *Bifidobacterium* in the infant gut is significantly lower than that previously reported, indeed, Western countries have reported *Bifidobacterium* abundances of more than 50% in the infant gut.^36,37^ However, other studies on the gut microbiota of Chinese infants have also demonstrated lower relative abundance of *Bifidobacterium*.^38–40^ This may be due to dietary and lifestyle differences between the different geographic locations or other factors related to genetic differences.^41^

Our results show that *Bifidobacterium* is among the dominant bacteria in the infant gut, and its relative abundance increased in a certain period in early life. The dominant *Bifidobacterium* in the maternal gut were *B. pseudocatenulatum* and *B. longum*, while the dominant *Bifidobacterium* in infants were *B. longum, B. pseudocatenulatum, B. dentium, B. breve* and *B. animalis* subsp. *lactis*. It has been reported that dietary intervention with non-digestible but fermentable carbohydrates can increase the abundance of *B. pseudocatenulatum*,^42^ which may explain the higher abundance of *B. pseudocatenulatum* in the maternal gut who generally consume more dietary fiber than infants. Previous studies have shown that the most prevalent *Bifidobacterium* species in vaginally-delivered infants were *B. breve, B. bifidum* and *B. longum* subsp. *infantis*,^43,44^ which was not consistent with our current results, but such discrepancies may be due to differences in living environment, diet and host genetics. For instance, it has been shown that bifidobacteria belonging to *B. longum* subsp. *infantis, B. bifidum* and *B. breve* taxa contain specific gene clusters encoding enzymes that are capable of hydrolyzing certain HMOs.^45–48^ In addition, the abundance of *B. breve* and *B. dentium* significantly increased in 42-day-old infant. However, the abundance of *B. bifidum* and *B. adolescentis* was lower in this study compared with previous reports, which might relate to the age of subjects.^36^ *B. dentium* has been reported to have greater tolerance against acid and bile salts, and aids the emulsification of lipids in breast milk,^49,50^ therefore, higher abundance of *B. dentium* may be associated with breast feeding.

Vertical transmission of gut bacteria from mother to their offspring is considered to be a pivotal route for microbiota establishment in newborns, although in-depth evaluation of this process has not been performed.^51–53^ In the current work, it was observed that some genera were dominant in infants but were less abundant in maternal samples which may be potentially vertical transmitted. Additionally, our results showed maternal gut may not be the only source for bacterial strains for the infant, such as *Bifidobacterium*. As observed in this study, not every mother-infant pair can be isolated from paired bifidobacterial. However, part of mother infant pairs can be isolated pairwise. To analyze whether the bifidobacteria in the infant intestine were transmitted from mother or not, we isolated bifidobacteria from all those samples and performed genomic comparison for each mother-infant pair shared species. ANI analysis of *Bifidobacterium* strains isolated from mother (during the last week of pregnancy) and infant fecal samples over the 42 day period showed that only *B. longum* subsp. *longum* strains from FGZ6 mother-infant pair had ANI values below 99%, indicating that these strains were not from the same source, but other isolates from the same mother-infant-pairs had the same origin.^54^ These results suggest that most of the *Bifidobacterium* strains shared by the same mother-infant-pair were homologous.^55^ But due to the change of gut environment, variance in the genomes of some strains may occur during the process of transmission.

Predicted results of carbohydrate utilization enzymes showed that the enzyme composition of *B. longum* subsp. *longum* strains from mother-infant-pairs FGZ6 and FGZ19 (not of the same source based on ANI analysis) were different in each pair. In particular, *B. longum* subsp. *longum* FGZ6-I-d7-M6 had more enzymes belonging to the CE10 family, which is involved in the utilization of oligosaccharides.^56^ Because of the different nutritional conditions within the infant and maternal gut environments, bacterial strains may be different at genome and functional levels. A study of the genome of *B. longum* subsp. *longum* strains isolated from subjects ranging from pre-weaning to centenarian showed that genes associated with carbohydrate transportation and metabolism in the infant-derived strains were significantly more abundant than those in adults and elders.^57^

Analysis of antibiotic resistance genes indicated that the abundance of resistance genes was different between strains pairs of FGZ19. In addition, the similar carbohydrate utilization gene profiles, antibiotic resistance genes, and high ANI values of the isolates of the same species from mother-infant-pairs, namely FGZ16 and FGZ36, suggest that they may be from the same sources. A study using MLST and AFLP to analyze the transmission of *B. longum* subsp. *longum* strains from mother to infant showed that strains from the same mother-infant-pair were monophyletic, and the strains from each family did not cluster.^53^

## Conclusion

The composition and relative abundance of the gut microbiota in early-life infants was significantly different from their mothers, whereby the infant gut microbiota was dominated by *Bifidobacterium*. Maternal and infant samples contained similar bifidobacterial profiles at the species level, but differed in their relative abundances, while the same bifidobacteria revealed different relative abundances in 7 and 42-day infant feces. Additionally, we provide evidence that bifidobacterial strains, such as *B. longum* subsp. *longum*, may transfer directly from mother to infant.

## Supplementary Material

Supplemental Material
