# Supplementary material for: Development of gut microbiota and bifidobacterial communities of neonates in the first 6 weeks and their inheritance from mother
Source: Gut Microbes. 2021 Apr 13;13(1):1908100. doi: 10.1080/19490976.2021.1908100 (PMC8049200; doi:10.1080/19490976.2021.1908100)

**Figure S1 LEfSe analysis of gut microbiota from mothers and infants**

(A) LEfSe analysis based on LDA between group 7-day-old infant and 42-day-old infant

(B) LEfSe analysis based on LDA between group 7-day-old infant and mother

(C) LEfSe analysis based on LDA between group 42-day-old infant and mother


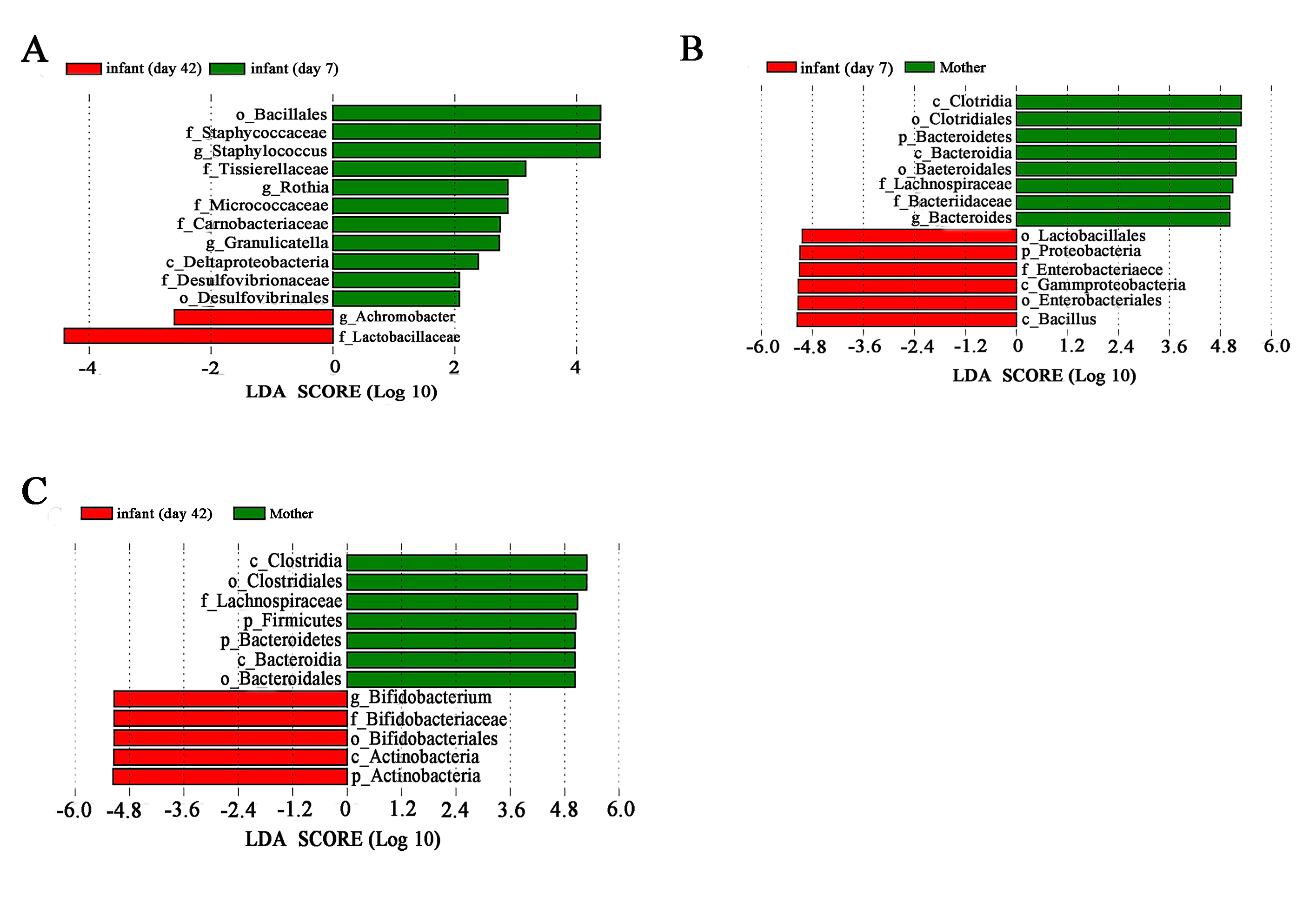


**Figure S2 Carbohydrate utilization enzymes in *Bifidobacterium* isolates from mother-infant pairs.**

The red bar signifies that the corresponding gene is present in *Bifidobacterium* isolated in this sample and black bar means absence.


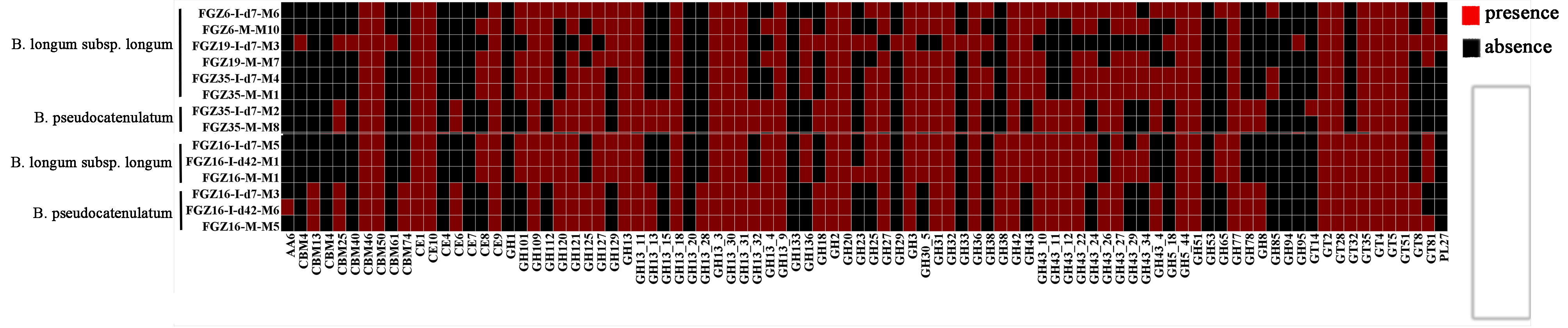


**Figure S3** **Predicted antibiotic resistance gene in Bifidobacterium isolates from mother-infant pairs.**

The red bar signifies that the corresponding gene is present in *Bifidobacterium* isolated in this sample and black bar means absence.


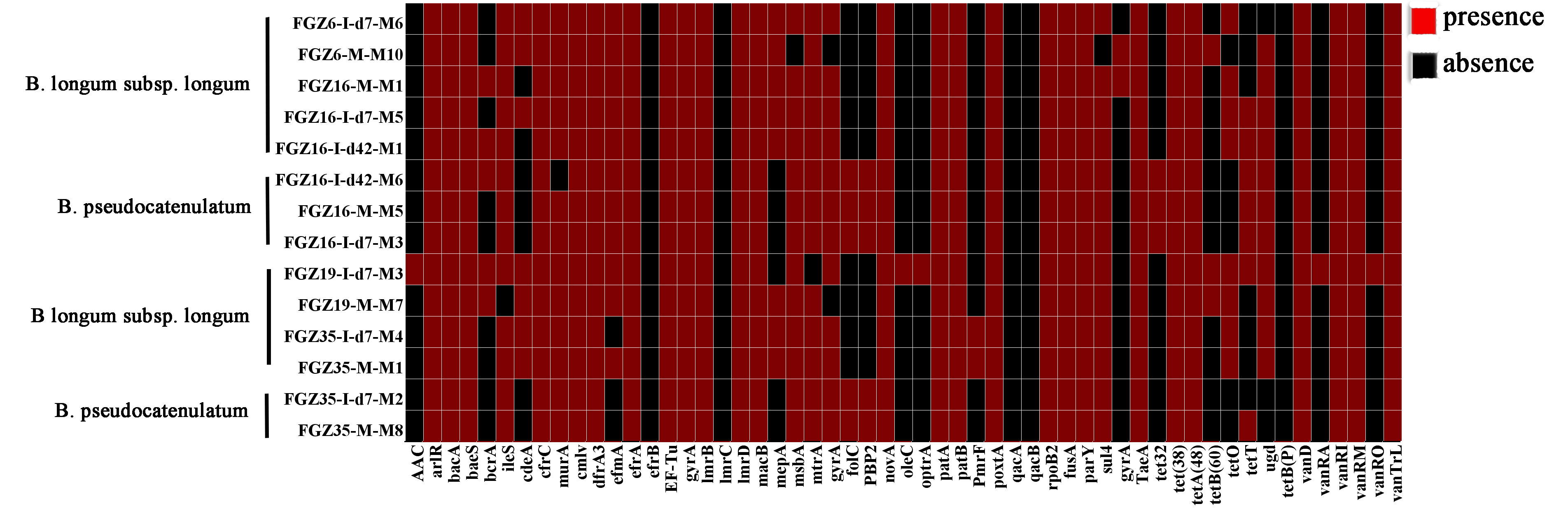

Supplement: Supplemental Material [file KGMI_A_1908100_SM1991.docx]
